# Study on Protection of Human Umbilical Vein Endothelial Cells from Amiodarone-Induced Damage by Intermedin through Activation of Wnt/*β*-Catenin Signaling Pathway

**DOI:** 10.1155/2021/8889408

**Published:** 2021-08-14

**Authors:** Yanhong Wang, Juanjuan Wang, Jia Yang, Jing Kang, Fuping Xue, Sijia Chang, He Ji, Haojing Zang, Xiaoshuang Zhou, Guiqin Wang, Weiping Fan, Xianyan Yan, Jinli Guo, Xiaojun Ren, Jihua Tian

**Affiliations:** ^1^Department of Microbiology and Immunology, Shanxi Medical University, Taiyuan, Shanxi 030001, China; ^2^Department of Intensive Care Unit, The Second Hospital of Shanxi Medical University, Taiyuan, Shanxi 030001, China; ^3^Department of Oncology, Xi'an Daxing Hospital, Xi'an, Shanxi 710016, China; ^4^Department of Nephrology, Shanxi Provincial Corps Hospital of Chinese People's Armed Police Forces, Taiyuan, Shanxi 030006, China; ^5^Department of Nephrology, Postdoctoral Workstation of Shanxi Provincial People's Hospital, The Affiliated People's Hospital of Shanxi Medical University, Shanxi Kidney Disease Institute, Taiyuan, Shanxi 030012, China; ^6^Department of Orthopedics, The Second Hospital of Shanxi Medical University, Taiyuan, Shanxi 030001, China; ^7^Department of Nephrology, The Affiliated Bethune Hospital of Shanxi Medical University, Shanxi Bethune Hospital (Shanxi Academy of Medical Sciences), Taiyuan, Shanxi 030032, China

## Abstract

Amiodarone (AM) is one of the most effective antiarrhythmic drugs and normally administrated by intravenous infusion which is liable to cause serious phlebitis. The therapeutic drugs for preventing this complication are limited. Intermedin (IMD), a member of calcitonin family, has a broad spectrum of biological effects including anti-inflammatory effects, antioxidant activities, and antiapoptosis. But now, the protective effects of IMD against amiodarone-induced phlebitis and the underlying molecular mechanism are not well understood. In this study, the aim was to investigate the protective efficiency and potential mechanisms of IMD in amiodarone-induced phlebitis. The results of this study revealed that treatment with IMD obviously attenuated apoptosis and exfoliation of vascular endothelial cells and infiltration of inflammatory cells in the rabbit model of phlebitis induced by intravenous infusion of amiodarone compared with control. Further tests in vitro demonstrated that IMD lessened amiodarone-induced endothelial cell apoptosis, improved amiodarone-induced oxidative stress injury, reduced inflammatory reaction, and activated the Wnt/*β*-catenin signal pathway which was inhibited by amiodarone. And these effects could be reversed by Wnt/*β*-catenin inhibitor IWR-1-endo, and si-RNA knocked down the gene of Wnt pathway. These results suggested that IMD exerted the protective effects against amiodarone-induced endothelial injury via activating the Wnt/*β*-catenin pathway. Thus, IMD could be used as a potential agent for the treatment of phlebitis.

## 1. Introduction

Amiodarone is one of the most commonly used antiarrhythmic drugs. It is used to control a wide spectrum of cardiac tachyarrhythmias ranging from premature ventricular and atrial contraction to sustained tachyarrhythmias, such as ventricular tachycardia and atrial fibrillation. It remains one of the most frequently prescribed antiarrhythmic medications; however, its use has been limited by multiple and serious side effects [1]. It can cause toxicity to organs such as the heart, lungs, thyroid gland, liver, eyes, skin, and nerves [2, 3]. It has been demonstrated that phlebitis is a common adverse reaction of peripheral intravenous administration of amiodarone, with an incidence of up to 85% [4]. Although phlebitis is a kind of aseptic inflammation of veins, the absence of appropriate interventions can increase the pain of patients, prolong the length of hospital stay [5, 6], and, in severe cases, lead to local skin and soft tissue necrosis and thrombophlebitis [7, 8]. Furthermore, few desired agents have been used in clinical treatment [9], and effective interventions for phlebitis remain seriously inadequate. At present, the mechanism of phlebitis induced by amiodarone has not been reported. It has been found that endothelial cell apoptosis, oxidative stress injury [10, 11], and inflammation may be involved in the formation of phlebitis.

Intermedin (IMD), also known as adrenomedullin-2 (AM2), is a recently identified peptide that belongs to the calcitonin gene-related peptide (CGRP) family [12, 13]. IMD has many biological functions including anti-inflammatory [14, 15], antioxidative stress [16], antiapoptosis [17], and participation in vascular remodeling [18]. The Wnt signaling pathway is one of the evolutionarily conserved pathways. It is ubiquitous in organisms and is widely involved in a variety of physiological processes. Some studies have suggested that the Wnt signaling pathway is associated with inflammation and oxidative stress injury. Our previous study confirmed that this pathway is also closely related to the protective effect of IMD on HUVEC injury induced by ischemia-reperfusion. It has been reported that IMD promotes the proliferation of hepatocellular carcinoma cells by activating Wnt signaling pathway [19]. The multiple properties may contribute to the potential benefit of IMD to patients suffering from phlebitis. This study investigated the protective effects of IMD against vascular endothelial cell injury induced by amiodarone in HUVECs and in a rabbit model of auricular phlebitis.

## 2. Materials and Methods

Amiodarone was purchased from Sanofi (Shanghai, China). Intermedin (IMD) (purity≧98%) was obtained from Phoenix Pharmaceutical Inc. (Belmont, CA, USA). Inhibition of the Wnt/*β*-catenin signaling pathway (IWR) was purchased from MedChemExpress (NJ, USA). Dulbecco's modified Eagle's medium (DMEM) and fetal bovine serum (FBS) were acquired from Solarbio (Beijing, China). Primary antibodies for Bcl-2, Bax, caspase-3, IL-1*β*, IL-6, TNF-*α*, IL-10, and *β*-actin were acquired from Proteintech Group (Chicago, USA). The commercial kits for measuring reactive oxygen species (ROS), lactate dehydrogenase (LDH), superoxide dismutase (SOD), glutathione peroxidase (GSH-Px) and catalase (CAT) activity, cell apoptosis (V-FITC/PI), and mitochondrial membrane potential (*ΔΨ*m) were purchased from Nanjing Jiancheng Bioengineering Institute (Nanjing, China). The *β*-catenin siRNA recombinant plasmid vectors were purchased from Sangon Biotech (Shanghai, China). Lipofectamine 2000 was acquired from Invitrogen (MA, USA).

### 2.1. Animal Model

Specific pathogen-free Japanese white rabbits (weights: 1.8–2.2 kg), obtained from Shanxi Medical University Animal Experimental Center, were housed in groups of three per standard cage, on a 12 h light/dark cycle; and air temperature was maintained at 26 ± 1°C. Experiments were implemented following Chinese animal welfare guidelines and were approved by the institutional ethics committee. All rabbits were fed in different cages taking drink freely.

Eighteen rabbits were randomly divided into three groups: normal control group (Control), amiodarone group (AM), and intermedin group (IMD). The animals in the AM group were intravenously infused with amiodarone (1.8 mg/mL for 12 h) via the peripheral vein of the right ear. Rabbits in IMD were pretreated with IMD (0.1 mg/kg in 2 mL of saline was injected 1 h before the amiodarone intravenous infusion). The rabbits in the Control group were challenged with the saline solution.

### 2.2. Cell Culture and Treatment

Human umbilical vein endothelial cells (HUVECs) were obtained from the Cell Bank of the Chinese Academy of Sciences (Shanghai, China) and were cultured in DMEM containing 10% fetal bovine serum at 37°C with 5% CO_2_. Cells used for all experiments were all in the exponential phase of the growth stage. In order to study the cytoprotective effects of IMD, HUVECs were divided into four groups for all experiments: Control group, untreated; AM group, amiodarone (30 *μ*mol L^−1^); IMD group, amiodarone (30 *μ*mol L^−1^)+IMD (20 ng mL^−1^) [20]; and IWR group, amiodarone (30 *μ*mol L^−1^)+IMD (20 ng mL^−1^)+IWR (10 *μ*mol L^−1^). IMD and IWR were applied 2 h prior to treatment with amiodarone.

### 2.3. Cell Transfection

Lipofectamine 2000 was used to transfect the corresponding plasmids into cells based on the manufacturer's instruction. Transfected cells were collected for mRNA and protein expression analysis after 24 incubation. After that, samples were divided into five groups: si NC group (blank carrier transfected), si NC+AM group (blank carrier transfected+amiodarone), si NC+IMD group (blank carrier transfected+amiodarone+IMD), siRNA+AM group (*β*-catenin siRNA transfected+amiodarone), and siRNA+IMD group (*β*-catenin siRNA transfected+amiodarone+IMD).

### 2.4. Cell Viability Assay

Cell viability was determined using the CCK-8 assay. In brief, 5 × 10^3^ cells were seeded into 96-well culture plates allowed to adhere overnight, and then, the cells were changed to fresh medium in each group. After incubation for 24 h, CCK-8 was added to each well for 4 h, and the absorbance was measured at 450 nm by a microplate reader (C-5000, Institute of Biophysics, Chinese Academy of Sciences, China). Cell viability in vehicle control groups was considered 100%. Each assay was carried out at least in triplicate.

### 2.5. Western Blotting Analysis

After the designated treatments, protein was extracted from the cells and the concentration was measured by a BCA Protein Assay Kit (KeyGEN Biotech, China). Equal amounts of protein (30 *μ*g) were separated on SDS-PAGE gels and transferred onto PVDF membranes. After blocking with 5% nonfat milk for 2 h at room temperature and washing with Tris-Buffered Saline-Tween 20 solutions (TBST) for 3 times in 10 min, the membranes were incubated overnight at 4°C with the following primary antibodies: anti-Bax (1 : 5000),anti-Bcl-2 (1 : 1000), anti-caspase-3 (1 : 1000), anti-IL-1*β*, anti-IL-6, anti-TNF-*α*, anti-IL-10, and anti-*β*-actin (1 : 5000). Membranes were washed as described previously and incubated with HRP-conjugated goat anti-rabbit IgG secondary antibodies for 2 h at room temperature. Blots were visualized by the ECL chemiluminescence system.

### 2.6. Real-Time PCR Analysis

Total RNA was isolated by Trizol and was reverse-transcribed to cDNA by Super Script II (Invitrogen). Target genes were amplified by SYBR Green I real-time PCR (RT-PCR). Primer pairs and amplification reaction parameters are listed in Table 1. GAPDH was used as an internal reference.

### 2.7. Release of Lactate Dehydrogenase (LDH) and Measurement of Superoxide Dismutase (SOD), Glutathione Peroxidase (GSH-Px), and Catalase (CAT) Activity

HUVECs were cultured in 6-well plates. After 24 h of various treatments, the release of LDH and SOD, GSH-Px, and CAT activity were determined by a commercial kit according to the manufacturer's instructions. The release amount of LDH is calculated according to the following formula [21]:

(1) $$\begin{array}{c} \text{LDH released}\%=\frac{\text{LDH activity in the medium}}{\text{LDH activity in the medium}+\text{in the cell}}\times 100\%. \end{array}$$

The LDH release rate and SOD, GSH-Px, and CAT activity in vehicle control groups were considered 100%. All the experiments were repeated for three times.

### 2.8. Measurement of Reactive Oxygen Species (ROS)

After treatment, HUVECs were harvested and incubated with 10 *μ*M membrane-permeable fluorogenic dye DCFH-DA for 20 min in FBS-free medium in the dark. The cells were slightly shaken every 5 min. After washing the cells with a serum-free culture medium, samples were analyzed for the fluorescence by flow cytometry. All procedures were repeated for five times.

### 2.9. Wound Healing Assay

Cells were seeded into 6-well plates and incubated. When cells reached 80% confluence, monolayer cells were scraped off by a 10 *μ*L sterile pipette tip. After washed with PBS for 3 times, cultured in an FBS-free culture medium. A phase-contrast microscope was used to monitor cells at the borders of the scratches. The degree of scratch healing was observed, and images in each group were captured at 0 h and 24 h. The area of wound healing in vehicle control groups was considered 100%.

### 2.10. Detection of Mitochondrial Membrane Potential

The mitochondrial membrane potential assay kit with JC-1 (Beyotime, China) was used to analyze changes of mitochondrial membrane potential (*ΔΨ*m). At the end of the drug treatment, cells were incubated with JC-1 at 37°C for 20 min in the dark, then washed twice with JC-1 working solution. Fluorescence intensity was observed with a confocal microscopy.

### 2.11. Detection of Cell Apoptosis

The apoptotic cells were measured by Annexin V-FITC/PI assays. After 24 h of various treatments, HUVECs were treated with an Annexin V-FITC/PI apoptosis kit according to the manufacturer's manual. In brief, HUVECs (1-5 × 10^5^) treated with drug were collected and resuspended in Annexin V-FITC binding buffer (500 *μ*L) after centrifugation for 5 min. With supplementation of Annexin V-FITC (5 *μ*L) and propidium iodide (PI) (5 *μ*L), cells were incubated at room temperature for 10 min in the dark. Apoptosis was measured by flow cytometry. The cell apoptotic rate in vehicle control groups was considered 100%. All procedures were repeated for three times.

### 2.12. Hematoxylin & Eosin (H&E) Stain

The isolated peripheral vein of the ear from different groups was fixed in 10% buffered formalin and embedded in paraffin, and sections (6 *μ*m) were prepared and stained with H&E. H&E stain was performed according to the standard procedure. Sections were observed and imaged under a light microscope (Olympus EX51, Japan).

### 2.13. TUNEL Assay

TUNEL assay (In-Situ Cell Death Detection Kit, POD; Roche) was used to evaluate the apoptotic cells in the rabbit ear veins. The TUNEL assay was performed according to the producer's protocol. Briefly, tissue sections were incubated with proteinase K solution (10–20 *μ*g/mL) for 30 min and then washed twice in PBS. Next, tissue sections were incubated with 50 *μ*L of the TUNEL reaction mixture at room temperature for 60 min in the dark. After washing again in PBS, 50 *μ*L of the Converter-POD was added to the sections for 30 min and followed by 3-amino-9-ethyl carbazole (AEC). Sections were then counterstained with hematoxylin. The brown nuclei of the cells were considered apoptotic testicular cells.

### 2.14. Statistical Analysis

All data represented as the mean ± SD. Statistical significance was analyzed by one-way ANOVA and assessed by GraphPad Prism v8. When *p* values < 0.05, it was considered to be statistically significant.

## 3. Results

### 3.1. The Effects of IMD in the Migration and Apoptosis of HUVECs

**Figure 1:**
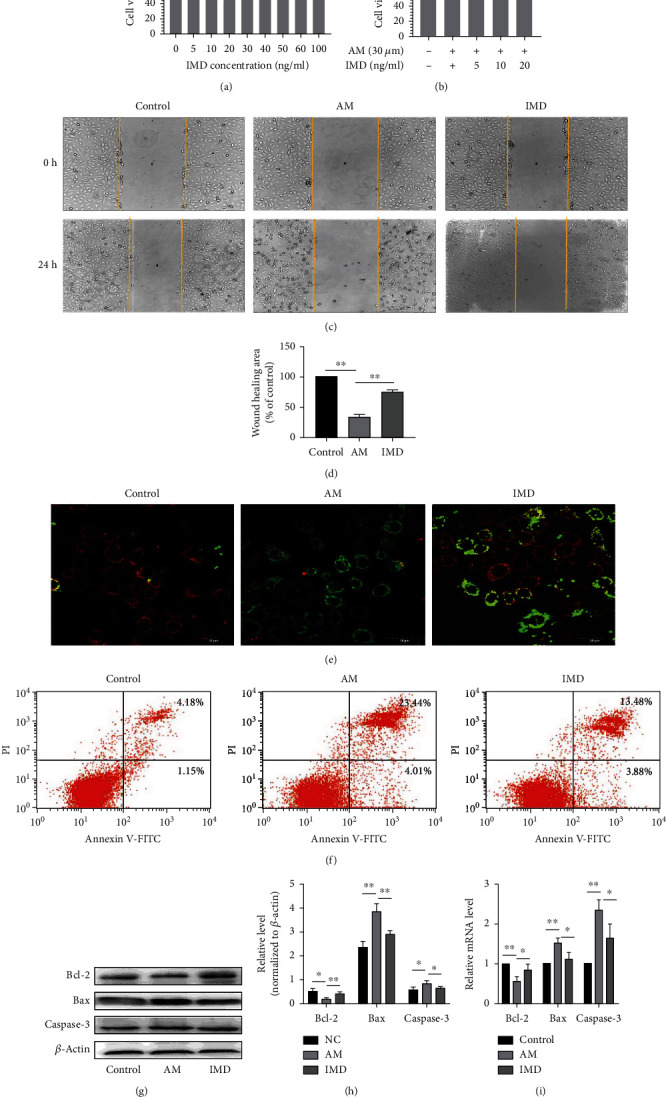
IMD protects HUVECs from amiodarone-induced proliferation, migration, and apoptosis. (a) HUVECs were treated with different concentrations of IMD, and cell viability was assessed via CCK-8 assay. (b) HUVECs were pretreated with different concentrations of IMD, followed by the stimulation of amiodarone; then, the cell viability was assessed via CCK-8 assay. (c, d) After pretreatment with IMD (20 ng/mL) for 2 h, HUVECs were incubated with amiodarone (30 *μ*mol/L) for 24 h. The wound healing assay was performed to test cell migration. (e) The *ΔΨ*m was detected by the JC-1 assay. (f) Flow cytometry analysis was performed to determine the apoptosis rate of each group. (g, h) The protein expressions Bcl-2, Bax, and caspase-3 were detected by western blotting analysis. (i) The mRNA levels of Bcl-2, Bax, and caspase-3 were detected by RT-PCR. Data was presented as the mean ± standard deviation (SD) (*n* = 3; ^∗^*p* < 0.05 and ^∗∗^*p* < 0.01 vs. control).

Firstly, to determine the optimal concentration and duration of administration, we examined the cell viability of HUVECs under amiodarone stimulation using the CCK-8 assay. HUVECs were treated with different concentrations of amiodarone in a range of 5–60 *μ*mol/L for 24 h, and the results showed that cell viability dropped to about 65% (*p* < 0.01) at 30 *μ*mol/L of amiodarone (supplementary figure a). Therefore, 30 *μ*mol/L of amiodarone was used in the following experiments. Next, we exposed HUVECs to 30 *μ*mol/L amiodarone for different times, and the results showed that cell viability decreased in a time-dependent manner (supplementary figure b). To demonstrate that IMD did not damage HUVECs, we treated HUVECs with different concentrations of IMD in the range of 5-100 ng/mL for 24 h, followed by the cell viability assay. As shown by the results of the CCK8 assay, the viability of cells treated with IMD was not influenced (Figure 1(a)). To determine whether IMD can improve amiodarone-induced damage and assess the optimum concentration for treatment, we treated HUVECs cultured under amiodarone conditions with different concentrations of IMD (5–20 ng/mL). The result is that the protective effect of IMD on HUVEC was related to its concentration. At a concentration of 20 ng/mL of IMD, the cell viability raised up to 92.62%, while the AM group was 75% (*p* < 0.001, Figure 1(b)); thus, this concentration was used for the following experiments. The migration of HUVECs plays an important role in the repair process after vascular injury. So, we evaluated the migration capability of HUVECs by the wound healing assay. It was observed that amiodarone inhibits HUVEC's movement and IMD promotes HUVEC's migration (Figures 1(c) and 1(d)). The decrease of ΔΨm is a landmark event in the early stage of apoptosis. The results showed that, compared with the Control group, ΔΨm decreased in the amiodarone-treated group, while pretreatment with IMD could improve this situation (Figure 1(e)). Moreover, the results of the Annexin V-FITC/PI assay showed that IMD significantly decreased the apoptosis rate of cells induced by amiodarone (Figure 1(f)). The quantitation result demonstrated that the percentage of apoptosis cells changed from 27.45% in the AM group to 17.36% in the IMD group (*p* < 0.01). Next, we assessed the expression levels of apoptosis-related molecules (Bcl-2, Bax, and caspase-3) to further determine the effects of IMD on apoptosis. The results showed that IMD can significantly improve changes in Bcl-2, Bax, and caspase-3 expression induced by amiodarone at both protein and mRNA levels (Figures 1(g)–1(i)).

### 3.2. IMD Protects HUVECs from Amiodarone-Induced Inflammation and Oxidative Stress

**Figure 2:**
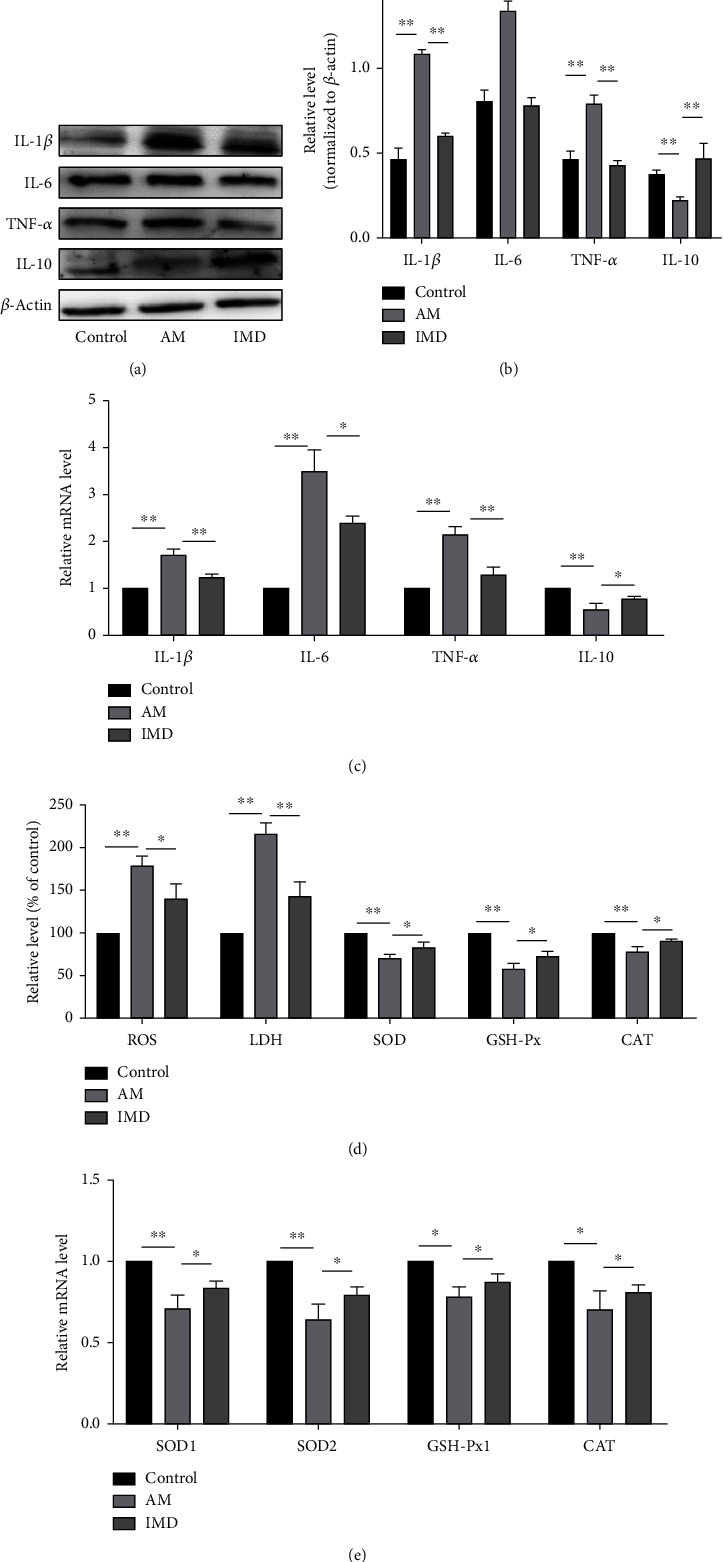
IMD protects HUVECs from amiodarone-induced inflammation and oxidative stress. (a, b) The expression of proinflammatory cytokines, including IL-1*β*, IL-6, and TNF-*α*, and anti-inflammatory IL-10 was detected by western blotting. (c) The mRNA levels of IL-1*β*, IL-6, TNF-*α*, and IL-10 were detected by RT-PCR. (d) The production of ROS, the release of LDH, and the vitality of SOD, GSH-Px, and CAT were detected using the standard assay. (e) The expression of antioxidant genes (SOD1, SOD2, GSH-Px1, and CAT) was detected by RT-PCR. Data was presented as the mean ± standard deviation (SD) (*n* = 3; ^∗^*p* < 0.05, ^∗∗^*p* < 0.01 vs. control).

It was reported that inflammatory cytokines increased significantly in the vinorelbine-induced phlebitis rabbit model [22]. To elucidate the effects of IMD on inflammation induced by amiodarone in HUVECs, we detected the expression of inflammatory cytokines by western blotting and RT-PCR. As shown by the results, compared with AM group, the IMD group showed significantly upregulated expression of anti-inflammatory cytokines (IL-10) and downregulated expression of proinflammatory cytokines (IL-6, IL-1*β*, and TNF-*α*) (Figures 2(a)–2(c)). To evaluate whether IMD protects HUVECs from amiodarone-induced oxidative stress, we detected the ROS level, the LDH release rate, and the activity of SOD, GSH-Px, and CAT after pretreatment with IMD. HUVECs subjected to amiodarone exhibited a significant increase in the ROS level and the LDH release rate and a noticeable drop in the activity of antioxidant enzymes (SOD, GSH-Px, and CAT). But IMD treatment substantially increased antioxidant enzyme activity and decreased ROS level and LDH release rate (Figures 2(d) and 2(e)).

### 3.3. IMD Activates the Wnt/*β*-Catenin Signaling Pathway

**Figure 3:**
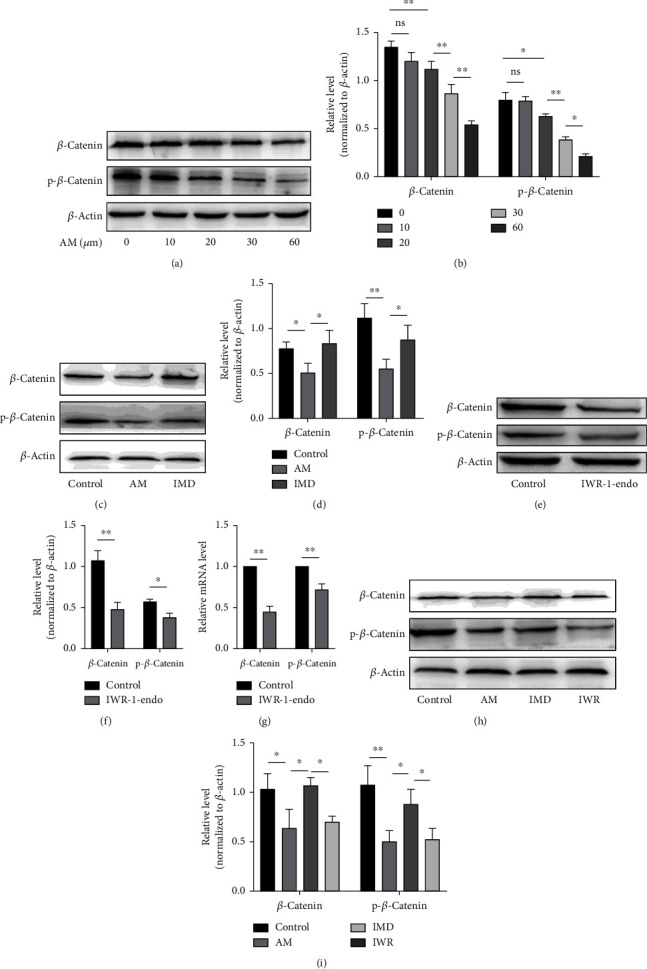
IMD activates the Wnt signaling pathway. (a, b) After treatment with different concentrations of amiodarone, the levels of *β*-catenin and p-*β*-catenin in HUVECs were determined by western blotting. (c, d) HUVECs were pretreated with IMD (20 ng/mL) for 2 h and then exposed to amiodarone (30 *μ*mol/L) for 24 h. The protein expression of *β*-catenin and p-*β*-catenin was detected by western blotting. (e–g) HUVECs were pretreated with IWR (10 *μ*mol/L) for 2 hours and then stimulated with amiodarone for 24 hours, and the expression levels of *β*-catenin and p-*β*-catenin were detected by western blotting. The mRNA levels were detected by RT-PCR. (h, i) After pretreatment with IMD (20 ng/mL) and IWR (10 *μ*mol/L) for 2 h, HUVECs were incubated with amiodarone for 24 h. The expression of *β*-catenin and p-*β*-catenin was detected by western blotting. Data was presented as the mean ± SD (*n* = 3; ^∗^*p* < 0.05 and ^∗∗^*p* < 0.01 vs. control).

We further revealed the related mechanism, after we confirmed the protective effect of IMD on HUVECs exposed to amiodarone. We investigated the relationship between IMD and Wnt pathways by pretreating HUVECs with IMD followed by stimulation with amiodarone. The results of western blotting and RT-PCR showed that amiodarone inhibited the expression of *β*-catenin and p-*β*-catenin, which is an important downstream factor of the Wnt signaling pathway, and the inhibition of amiodarone was increasingly apparent with increasing concentrations. (Figures 3(a) and 3(b)). Importantly, compared with the AM-treated group, IMD activated the Wnt signaling pathway, as evidenced by the increased phosphorylation level of *β*-catenin (Figures 3(c) and 3(d)). To further evidence that IMD exerts its protective effect through activating the Wnt signaling pathway, we used IWR to block the Wnt pathway. Indeed, the IWR can significantly suppress the phosphorylation level of *β*-catenin (Figures 3(e) and 3(g)). Moreover, pretreatment with IWR inhibited the IMD-induced upregulation of *β*-catenin phosphorylation levels (Figures 3(h) and 3(i)).

### 3.4. Blockade of Wnt/*β*-Catenin Signaling Pathway Inhibits the Protective Effects of IMD in HUVECs

**Figure 4:**
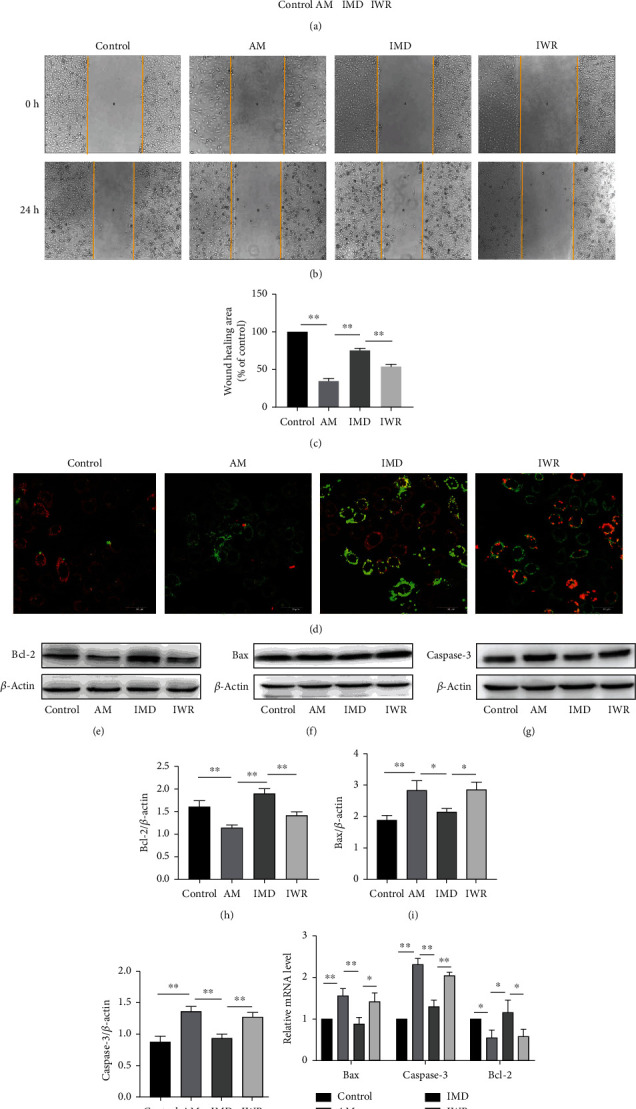
IWR abolishes the protective effects of IMD in HUVECs. HUVECs were pretreated with IMD (20 ng/mL) and IWR (10 *μ*mol/L) for 2 h and then incubated with amiodarone for 24 h. (a) Cell viability was assessed via CCK-8 assay. (b, c) The wound healing assay was performed to test cell migration. (d) The *ΔΨ*m was detected by the JC-1 assay. (e–j) The protein expressions of Bcl-2, Bax, and caspase-3 were detected by western blotting analysis. (k) The mRNA levels of Bcl-2, Bax, and caspase-3 were detected by RT-PCR. Data was presented as the mean ± SD (*n* = 3; ^∗^*p* < 0.05 and ^∗∗^*p* < 0.01 vs. control).

**Figure 5:**
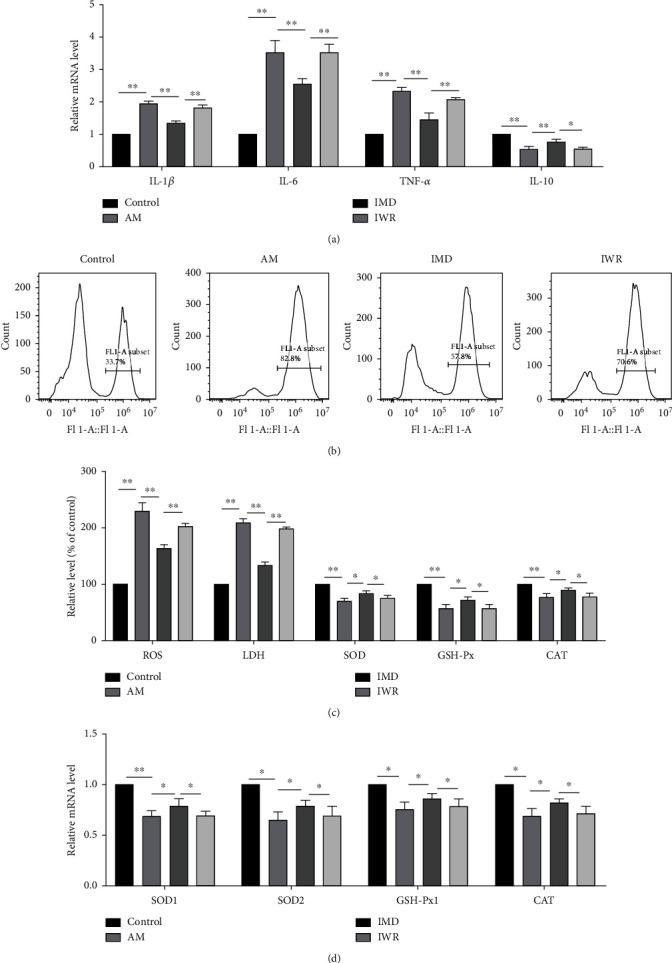
Effects of IWR on the protective effect of IMD on amiodarone-induced inflammation and oxidative stress injury to HUVECs. HUVECs were pretreated with IMD (20 ng/mL) and IWR (10 *μ*mol/L) for 2 h and exposed to amiodarone (30 *μ*mol/L) for 24 h. (a) Detection of expression of inflammation-related genes by RT-PCR experiment. (b, c) The kit detected the production of ROS, the release of LDH, and the vitality of SOD, GSH-Px, CAT. (d) Detection of expression of antioxidant genes by RT-PCR experiment. Data was presented as the mean ± SD (*n* = 3; ^∗^*p* < 0.05 and ^∗∗^*p* < 0.01 vs. control).

**Figure 6:**
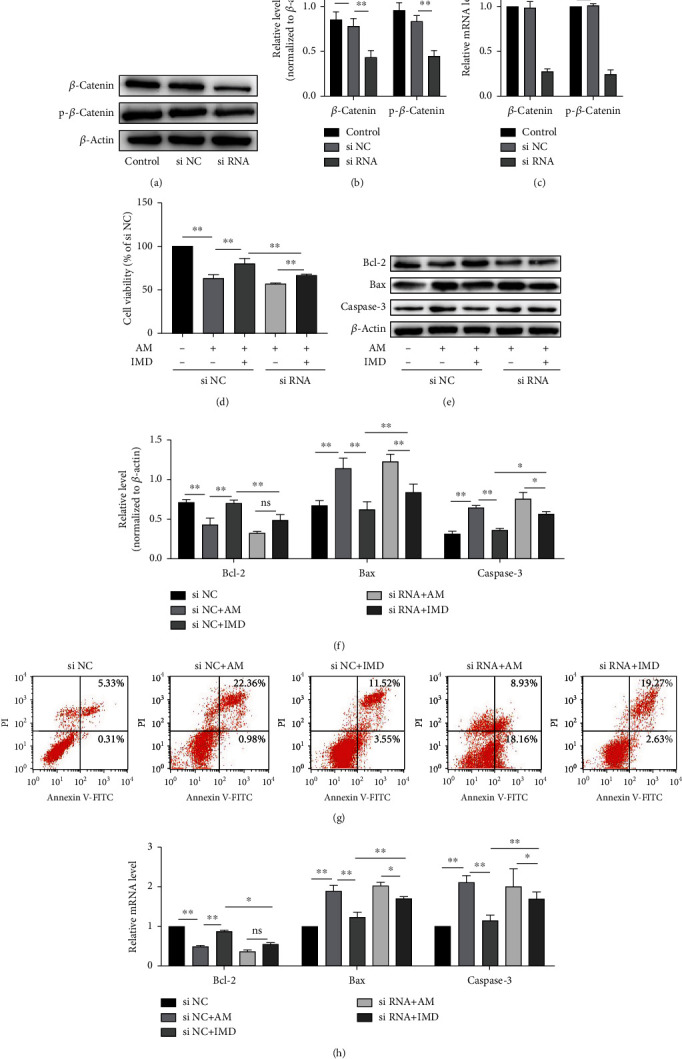
Wnt/*β*-catenin siRNA transfection in HUVECs inhibits the protective effects of IMD. The siRNA was used to specifically knock down the *β*-catenin in HUVECs, and the blank carrier was used as a negative control. After transfection, cells were divided into five groups: si NC group, si NC+AM group, si NC+IMD group, siRNA+AM group, and siRNA+IMD group. (a–c) The efficiency of *β*-catenin knockdown by its siRNA in HUVECs was assessed by western blotting and RT-PCR. (d) Cell viability was assessed via CCK-8 assay after transfection. (e, f) The protein expressions of Bcl-2, Bax, and caspase-3 were detected by western blotting analysis. (g) Flow cytometry analysis was performed to determine the apoptosis rate of each group. (h) The mRNA levels of Bcl-2, Bax, and caspase-3 were detected by RT-PCR. Data was presented as the mean ± standard deviation (SD) (*n* = 3; ^∗^*p* < 0.05 and ^∗∗^*p* < 0.01 vs. control).

**Figure 7:**
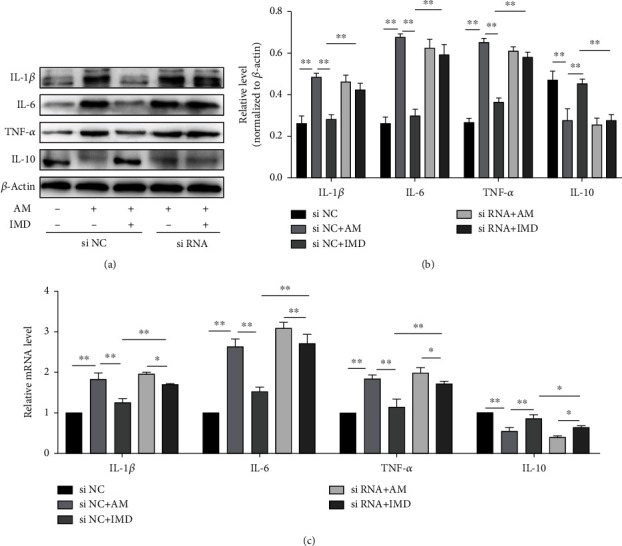
Wnt/*β*-catenin siRNA transfection in HUVECs inhibits the protective effects of IMD. (a, b) The expression of proinflammatory cytokines, including IL-1*β*, IL-6, and TNF-*α*, and anti-inflammatory IL-10 were detected by western blotting. (c) The mRNA levels of IL-1*β*, IL-6, TNF-*α*, and IL-10 were detected by RT-PCR. Data was presented as the mean ± standard deviation (SD) (*n* = 3; ^∗^*p* < 0.05 and ^∗∗^*p* < 0.01 vs. control).

To further confirm the role of the Wnt/*β*-catenin pathway in mediating the protective effect of IMD against amiodarone, the Wnt pathway was blocked with the specific inhibitor IWR. HUVECs were pretreated with IMD and IWR then stimulated with amiodarone. Compared with the AM group, the IMD group could promote cell vitality and cell invasion, reduce apoptotic rate, and improve inflammatory damage and oxidative stress. But pretreatment with IWR attenuated the protective effects of IMD in HUVECs, including its anti-injury (Figures 4(a)–4(c)), antiapoptosis (Figures 4(d)–4(k)), anti-inflammatory (Figure 5(a)), and antioxidant stress effects (Figures 5(b)–5(d)). To further evaluate the relationship between IMD and Wnt pathway, we performed the si-RNA to specifically knock down the gene of Wnt pathway. The results of RT-PCR and western blotting showed that administration of 50 nM siRNA significantly suppressed the mRNA expression of Wnt/*β*-catenin compared to the scrambled siRNA (*p* < 0.0001) (Figures 6(a)–6(c)). Consistently, blocking the Wnt pathway by si-RNA also weakened the protective ability of IMD in HUVECs, including its anti-injury (Figure 6(d)), antiapoptosis (Figures 6(e)–6(h)), and anti-inflammatory (Figure 7). Taken together, the results suggested that IMD may exert its beneficial effect through the Wnt pathway.

### 3.5. Effects of IMD on Amiodarone-Induced Inflammation and Apoptosis in Phlebitis of Rabbits

**Figure 8:**
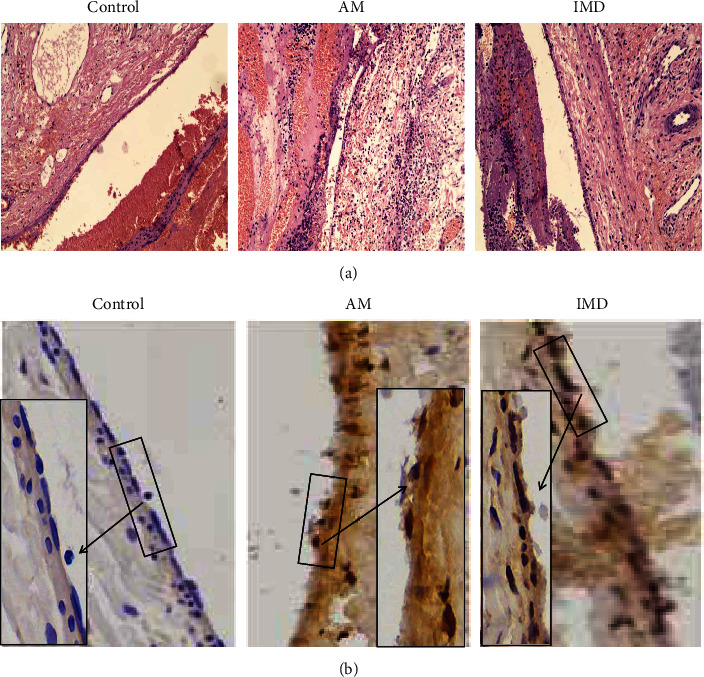
Effects of IMD on amiodarone-induced inflammation and apoptosis in phlebitis of rabbits. (a) Histopathological analysis of rabbit ear sections by HE staining (×100). (b) TUNEL assay was used to detect the apoptotic cells in the ear veins of rabbits (×100 and ×400). *n* = 6.

Intravenous infusion of amiodarone could cause pathological changes in the ear vein. The results of HE staining showed that after infusion of amiodarone, the structural integrity of the ear vein was damaged, the vascular endothelium was largely shed, and a large number of inflammatory cells were accumulated in vascular tissues. However, in the IMD group, an improvement in the pathological changes of phlebitis in the ear veins was observed, including a more intact structure of the ear vein and less infiltration of inflammatory cells in the IMD group (Figure 8(a)). The result of TUNEL staining (Figure 8(b)) showed decreased apoptotic cells in and around vascular tissues in the IMD-treated group compared with the AM-treated group.

## 4. Discussion

In the clinic, phlebitis often occurs when amiodarone is infused through the peripheral venous catheter. However, its underlying mechanisms remain unknown and there are no effective prevention measures. In this study, we investigated the amiodarone-induced damage to HUVECs, as observed by inducing inflammatory damage and oxidative stress injury, reducing cell viability, and promoting apoptosis, but IMD can improve these damages. Furthermore, amiodarone can inhibit activation of the Wnt signaling pathway, but IMD can activate it. Besides that, it showed that the protective effect of IMD on amiodarone-induced HUVEC injury is at least partially exerted through the Wnt signaling pathway. Additionally, we observed that intravenous infusion of amiodarone can cause damage to ear veins in rabbits; however, administration of IMD can alleviate this phenomenon.

Phlebitis is an inflammation of the blood vessel wall. The vascular endothelium cells are the most vulnerable site and play an important role in the development of phlebitis。HUVECs were selected for this present study to reveal the mechanism of amiodarone-induced phlebitis. We found that amiodarone can suppress HUVEC viability at a low concentration. HUVECs are very sensitive to amiodarone (LC50 = 40 *μ*M); this may be the reason why amiodarone causes phlebitis easily. Vascular endothelial cell apoptosis is closely related to phlebitis [23]. Importantly, the treatment of amiodarone at 30 *μ*M significantly induced the apoptosis of HUVECs (Supplementary Figure (c)). Several studies have demonstrated that oxidative stress played an important role in drug-induced endothelial cell injury. [22, 24, 25] Consistently, amiodarone promoted the production of inflammatory cytokines, including IL-6, IL-1*β*, and TNF-*α*, and inhibited the production of IL-10, an anti-inflammatory factor (Supplementary Figure (c) and (d)). Also, our results showed increased ROS production and LDH release rate and decreased antioxidant enzyme activity in amiodarone-treated HUVECs (Supplementary Figure (e)-(f)). The above results demonstrate that amiodarone can promote inflammation and induce oxidative stress injury in HUVECs. Therefore, anti-inflammatory and reducing oxidative stress injury may bring therapeutic benefit in amiodarone-induced phlebitis.

In the mouse model of sepsis, IMD decreases inflammatory conditions via downregulating CCR2 expression [14]. IMD can inhibit the gene expression of the proinflammatory cytokines (TNF-*α*, IL-6, and IL-1*β*) in LPS-induced rat testis inflammation [26]. Moreover, it has been proved that IMD can improve oxidative stress-induced injury in both diabetic rats [27] and IgA nephropathy mice [28]. However, whether IMD plays a protective role in phlebitis has not yet been reported. Therefore, we pretreated amiodarone-treated HUVECs with IMD. As we predicted, IMD can reduce amiodarone-induced oxidative stress damage in HUVECs, suggesting that the antioxidant activity of IMD contributes to its protective effects. Also, our results showed that amiodarone inhibited the viability of HUVECs at low concentrations, while an increase in cell viability was observed in the presence of IMD. Additionally, we found that pretreatment of IMD can improve the migration ability of HUVECs, which is important for the repair process in vascular injury. All these results imply the protective effects of IMD in endothelial cell injury. Apoptosis of vascular endothelial cells plays an important role in phlebitis. During bacterial sepsis, increased numbers of apoptotic ECs are detected in the pulmonary capillaries of a murine model of sepsis [29]. In LPS-induced liver sinusoidal EC injury, activation of caspase-3, a central apoptotic effector protease, is enhanced [23]. Importantly, previous studies have reported that IMD has a significant protective effect on neuronal damage and cardiomyocyte hypoxia injury by inhibiting apoptosis [30, 31]. In the present study, we found that IMD suppressed the amiodarone-induced apoptosis in both HUVECs and rabbit ear veins, which provides a new perspective of the mechanism underlying the protective role of IMD in phlebitis. Generally, oxidative stress is considered the main trigger of endothelial dysfunction. The continuous ROS generation leads to mitochondrial function, thereby causing cellular death [32]. Also, mitochondrial dysfunction contributes to cytochrome c translocation, which results in caspase-9 activation and subsequently causes cell apoptosis [33]. Indeed, we found that IMD can reduce amiodarone-induced oxidative stress damage, including the ROS production in HUVECs, suggesting that the antioxidant activity of IMD contributes to its protective effects. Phlebitis is a form of inflammation in the vein caused by intravenous infusion, presenting with the increased inflammatory responses of the injection site [34]. Previous studies have shown that IMD inhibits the inflammatory response in several diseases, such as orchitis [26] and uveitis [35]. In the present work, we found that IMD decreased the expression levels of TNF-*α*, IL-1*β*, and IL-6, which are well-known proinflammatory mediators that can enhance vascular injury, in amiodarone-treated HUVECs. Also, our results showed that IMD suppressed the infiltration of inflammatory cells in vascular tissue. All these results imply the protective effects of IMD in endothelial cell injury.

The Wnt signaling pathway is a highly complex pathway during the evolution of organisms and plays a vital role in cell growth, proliferation, differentiation, and maintenance of homeostasis. Under pathological conditions, the Wnt signaling pathway participates in various processes of diseases and plays different roles. The Wnt/*β*-catenin pathway is the most clearly studied pathway in Wnt signaling. Tang et al.'s investigation [36] demonstrated that a decrease in ROS levels is related to the activation of the Wnt/*β*-catenin pathway in a rat model of cerebral ischemia-reperfusion injury. Similar results were obtained in myocardial [37] and liver [38] ischemia-reperfusion injury models. It was reported that vitamin D could protect ROS accumulation in melanocytes induced by H_2_O_2_, and it works by activating the Wnt/*β*-catenin signaling pathway [39]. Application of Wnt signaling agonists increased the activity of SOD and GSH-PX and improved behavioral deficits in rats with Parkinson's disease [40]. The deficiency of this pathway gives rise to T cell-mediated immune response and incidence of AIH [41]. The Wnt/*β*-catenin plays an anti-inflammatory effect on pigs during bacterial infection [42]. Previous studies have reported that activating the Wnt/*β*-catenin signaling pathway could promote fibroblast growth and reduce apoptosis in rheumatoid arthritis models [43–45]. Importantly, we found that amiodarone caused inhibition of the Wnt signaling pathway in a concentration-dependent manner. However, IMD can protect HUVECs from amiodarone-induced injury through the activation of the Wnt/*β*-catenin signaling pathway, as observed by improved cell viability, ameliorated oxidative stress, reduced inflammation, and apoptosis. Moreover, using IWR, the specific inhibitor of the Wnt pathway can abolish the protective effect of IMD, suggesting that the Wnt/*β*-catenin pathway plays a key role in the action of amiodarone and IMD. Collectively, our results implied that the protective effect of IMD on the amiodarone-induced injury of HUVECs is mediated by the Wnt/*β*-catenin pathway.

**Figure 9:**
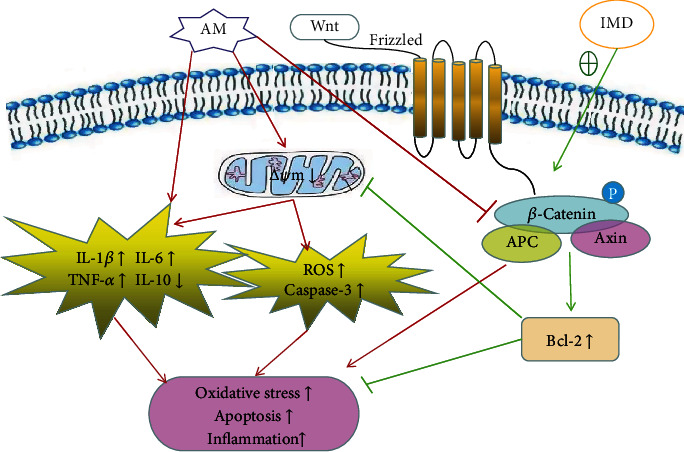
Schematic representation of mechanisms underlying the protective effects of IMD in attenuating amiodarone-induced oxidative stress, apoptosis, and inflammation in HUVECs.

Taken together, amiodarone-induced phlebitis is related to inflammation, oxidative stress, and apoptosis, which can be attenuated by treatment of IMD. In vitro, amiodarone inhibited the Wnt/*β*-catenin pathway in a concentration-dependent manner. On the contrary, IMD can activate the Wnt/*β*-catenin pathway, therefore ameliorated the amiodarone-induced damage in HUVECs. This at least partially verified that IMD plays a protective role against amiodarone-induced phlebitis by activating the Wnt/*β*-catenin pathway (Figure 9). Thus, IMD or potentially other agents that stimulate the Wnt/*β*-catenin pathway may be able to attenuate amiodarone-induced phlebitis. However, due to the small molecular weight and the fast metabolism rate of IMD, the way of IMD administration in protecting phlebitis still needs to be further explored.

## Figures and Tables

**Table 1:** Synthesis list of real-time PCR primers.

Gene name		Primer sequence 5′-3′
Bcl-2	F	GACTTCGCCGAGATGTCCAG
	R	GAACTCAAAGAAGGCCACAATC

Bax	F	CGAACTGGACAGTAACATGGAG
	R	CAGTTTGCTGGCAAAGTAGAAA

Caspase-3	F	GTGGAGGCCGACTTCTTGTATGC
	R	TGGCACAAAGCGACTGGATGAAC

IL-1*β*	F	GCCAGTGAAATGATGGCTTATT
	R	AGGAGCACTTCATCTGTTTAGG

IL-6	F	CACTGGTCTTTTGGAGTTTGAG
	R	GGACTTTTGTACTCATCTGCAC

IL-10	F	GTTGTTAAAGGAGTCCTTGCTG
	R	TTCACAGGGAAGAAATCGATGA

TNF-*α*	F	TGGCGTGGAGCTGAGAGATAACC
	R	CGATGCGGCTGATGGTGTGG

SOD1	F	CGAGCAGAAGGAAAGTAATGGA
	R	CACACCATCTTTGTCAGCAGTC

SOD2	F	CGTGACTTTGGTTCCTTTGAC
	R	ATTTGTAAGTGTCCCCGTTCC

GSH-Px1	F	AGTCGGTGTATGCCTTCTCG
	R	TCGTTCATCTGGGTGTAGTCC

CAT	F	GCCTTTGGCTACTTTGAGGTC
	R	GATGAAGAAAATGGGGGTGTTA

GAPDH	F	TGAACGGGAAGCTCACTGG
	R	TCCACCACCCTGTTGCTGTA

F: forward primer; R: reverse primer.

## Data Availability

The data used to support the findings of this study are included within the article.

## Supplementary Materials

Supplementary Materials Supplementary Figure: the effect of amiodarone on HUVECs.
