# Supplementary material for: Study on Protection of Human Umbilical Vein Endothelial Cells from Amiodarone-Induced Damage by Intermedin through Activation of Wnt/β-Catenin Signaling Pathway
Source: Oxid Med Cell Longev. 2021 Aug 14;2021:8889408. doi: 10.1155/2021/8889408 (PMC8382522; doi:10.1155/2021/8889408)
Supplement: Supplementary Materials — Supplementary Figure: the effect of amiodarone on HUVECs. [file 8889408.f1.zip › Supplementary Figure.doc]

Supplementary Figure: The effect of amiodarone on HUVECs. (a) HUVECs were treated with different concentrations of amiodarone, and cell viability was assessed via CCK-8 assay. (b) HUVECs were treated with the same concentration of amiodarone (30μmol/L) for a different time, and then cell viability was assessed via CCK-8 assay. HUVECs were treated with amiodarone (30μmol/L) for 24h: (c)The mRNA levels of Bcl-2, Bax, and caspase-3 were detected by RT-PCR. (d)The mRNA levels of IL-1β, IL-6, TNF-α and IL-10 were detected by RT-PCR. (e)The production of ROS, the release of LDH and the vitality of SOD, GSH-Px, and CAT were detected using the standard assay. (f) The expression of antioxidant genes (SOD1, SOD2, GSH-Px1, CAT) were detected by RT-PCR. Data was presented as mean ±SD. (n = 3, *p < .05, **p < .01 vs. control).
